# A bibliometric analysis of the knowledge related to mental health during and post COVID-19 pandemic

**DOI:** 10.3389/fpsyg.2024.1411340

**Published:** 2024-06-05

**Authors:** Lili Wang, Lingxiao Ye, Yanyan Jin, Xingying Pan, Xuesong Wang

**Affiliations:** ^1^Nursing Department, Ningbo Medical Center LiHuiLi Hospital, Ningbo, Zhejiang, China; ^2^Department of Emergency Medicine, Yuyao People’s Hospital, Medical School of Ningbo University, Ningbo, Zhejiang, China; ^3^College of Acupuncture-Moxibustion and Tuina, Hebei University of Chinese Medicine, Shijiazhuang, Hebei, China

**Keywords:** bibliometric, COVID-19 pandemic, mental health, depression, anxiety, CiteSpace, VOSviewers, R software

## Abstract

**Objective:**

COVID-19 led to a horrific global pandemic, with strict lockdowns and prolonged indoor stays increasing the risk of mental health problems, affecting people of different ages, genders, regions, and types of work to varying degrees. This study provides a bibliometric summary of the knowledge map related to mental health during and post COVID-19 pandemic.

**Methods:**

Publications related to mental health during and post COVID-19 pandemic were searched in the Web of Science Core Collection (WoSCC) database through March 19, 2024. After screening the search results, the literature included in the final was first quantitatively analyzed using GraphPad Prism software and then visualized using VOSviewer, CiteSpace, and R (the bibliometrix package).

**Results:**

The 7,047 publications from 110 countries were included, with the highest number of publications from China and the United States, and the number of publications related to mental health during and post the COVID-19 pandemic increased annually until 2023, after which it began to decline. The major institutions were University of Toronto, University of London, Harvard University, King’s College London, University College London, University of California System, University of Melbourne, Institut National De La Sante Et De La Recherche Medicale (Inserm), Mcgill University, and University of Ottawa; Frontiers in Psychiatry had the highest number of publications, and the Journal of Affective Disorders had the highest number of co-citations; 36,486 authors included, with Xiang, Yu-Tao, Cheung, Teris, Chung, Seockhoon published the most papers, and World Health Organization, Kroenke K, and Wang CY were the most co-cited; epidemiologically relevant studies on mental health related to COVID-19, and the importance of mental health during normalized epidemic prevention and control are the main directions of this research area, especially focusing on children’s mental health; “pandemic,” “sars-cov-2,” “epidemic,” “depression,” “coronavirus anxiety,” “anxiety,” “longitudinal,” “child,” “coronavirus anxiety,” “longitudinal,” “child,” and “coronavirus” are the top keywords in recent years.

**Conclusion:**

This comprehensive bibliometric study summarizes research trends and advances in mental health during and after the COVID-19 Pandemic. It serves as a reference for mental health research scholars during and after the COVID-19 pandemic, clarifying recent research preoccupations and topical directions.

## 1 Background

Coronavirus disease (COVID-19) is a contagious respiratory disease that World Health Organization declared to be an outbreak of a pandemic on 11 March 2020 (Van Doremalen et al., 2020; Zhu et al., 2020). Until March 10, 2023, the pandemic has had a catastrophic impact on health and mortality, with more than 670 million confirmed cases and 6.88 million deaths globally (Hopkins Johns Coronavirus Resource Center University, 2023; World Health Organization, 2023). In the initial 2 years of the pandemic, numerous countries implemented measures such as physical distancing, national blockades, and travel restrictions to control the spread of COVID-19 (Chtourou et al., 2020; National Health Commission of the People's Republic of China, 2023). The prolonged isolation, strict lockdowns, prolonged indoor stays, and the subsequent sequelae of the virus (Ercoli et al., 2021) (such as smell/taste disorders), all of which have been improved the risk of different forms of mental distress (de Lima et al., 2020; Pouso et al., 2021).

Psychosocial impacts follow each pathogen (Jones, 2020; Wang and Hong, 2020), SARS, smallpox, and hepatitis are classic examples of this dynamic, and the effects associated may not diminish over time (YuenMan, 2008), as is the case with COVID-19. Lockdown policies and other public health measures have been implemented to varying degrees around the world to prevent the spread of COVID-19 (Pierce et al., 2020; Turna et al., 2021), and continue to cause death and disability even after the World Health Organization has declared the end of the “emergency” phase (World Health Organization, 2020b), with varying degrees of impact across all age groups. Strict lockdowns and prolonged indoor stays increase the risk of depression, anxiety, and other mental health issues. The mental distress caused by the pandemic has affected people of different ages, genders, regions, and types of work to varying degrees. Studies have shown that young people have elevated levels of anxiety and depression and a significantly increased risk of suicide and self-harm (Holladay et al., 2022). Females are more severely affected than males, and individuals with pre-existing conditions are more likely to experience mental disorders (Özdin and Bayrak, 2020). Cluster analysis of symptoms in different regions showed that individuals in high-risk areas exhibited higher levels of anxiety and sleep disorders compared to low-risk areas (Wei et al., 2020). The global pandemic has had a significant impact on the mental health of workers across all sectors, with frontline health workers being particularly affected (World Socialist Web Site, 2020). According to studies conducted in China, approximately one-third of medical personnel experienced insomnia during this period, with severe insomnia rates reaching as high as 26.67% (Wu and Wei, 2020), and a psychological abnormality rate of 14.5% (Cai et al., 2020).

Bibliometrics allows the quantitative analysis of various knowledge carriers, and the measurement objects mainly include authors, keywords, journals, countries, institutions, references, etc., which allows the construction of knowledge maps (Wang et al., 2019; Ke et al., 2020). Commonly used bibliometric tools include CiteSpace (Synnestvedt et al., 2005), VOSviewer (Yeung et al., 2020), the R software (bibliometrix package) (Li et al., 2020), and HistCite (Lu et al., 2020). With the increase in COVID-related publications, several COVID-related bibliometric publications have been reported, such as COVID-19 with other diseases (Wei et al., 2022), mental retardation (Ying et al., 2022), suicidal behavior (Grover et al., 2021), depression disorders (Al-Jabi, 2021), and sleep disorders (Sun et al., 2024). Therefore, this study aimed to conduct a bibliometric analysis of publications related to Mental Health During and Post COVID-19 Pandemic, comprehensively and accurately capture the current state of development of the research topic, historical trends, and forecast future directions by analyzing key points.

## 2 Methods

### 2.1 Search strategy

Due to the limitations of bibliometric software Citespace and VOSviewer software, merging multiple databases (especially in Chinese and English) is impossible. At the same time, the Web of Science Core Collection (WoSCC) is an authoritative academic database covering relatively high-quality academic journals, which is a reliable source of data for conducting bibliometrics It is a reliable data source for bibliometric analysis. The WoSCC was used to search the related publications, retrieved until March 19, 2024, and the detailed search formula as follows: (“mental health” OR “mental illness” OR “mental illnesses” OR “mental disorder” OR “mental disorders” OR anxiety OR anxious OR depression OR depressive) AND (COVID-19 OR SARS-CoV-2 OR coronavirus OR 2019 nCoV). We restricted the publication types to “article” and “review.”

### 2.2 Data analysis

We performed a quantitative analysis of the number of publications using GraphPad 9.0.3 software; the publications retrieved from the database were visualized using VOSviewer (1.6.18), CiteSpace (6.1.R1), and R software (bibliometrix package, 3.2.1).

First, we quantitatively analyzed the annual number of publications and plotted bar charts using Graphpad 9.0.3 to obtain the overall research trends;

Then, VOSviewer software was used to visualize different measurement objects such as countries, institutions, journals, co-cited journals, authors, co-cited authors, and so on, to obtain the relationship between different measurement objects; each node represents a measurement object in the visual network map created by VOSviewer; while node sizes and colors denote the number and types of these items, respectively; the thickness of the lines between nodes reflects the strength of cooperation or co-citation between different measurement objects (Arruda et al., 2022; Shen et al., 2022);

Next, we plotted a dual-map overlay of journals using CiteSpace (version 6.1.R1) (developed by Prof. Chen C). This overlay reveals the overall flow relationship between journal cites and cited, as well as identifying the top 10 references with strong citation bursts using CiteSpace (Chen et al., 2012);

Finally, we used the R package (bibliometrix, version 3.2.1)^1^ to draw Trending Topics and Tree maps for future research direction prediction (Aria and Cuccurullo, 2017);

The journal partitions and impact factors used in this study were obtained from the Journal Citation Report 2020.

## 3 Results

### 3.1 Quantitative analysis of publication

**Figure 1:**
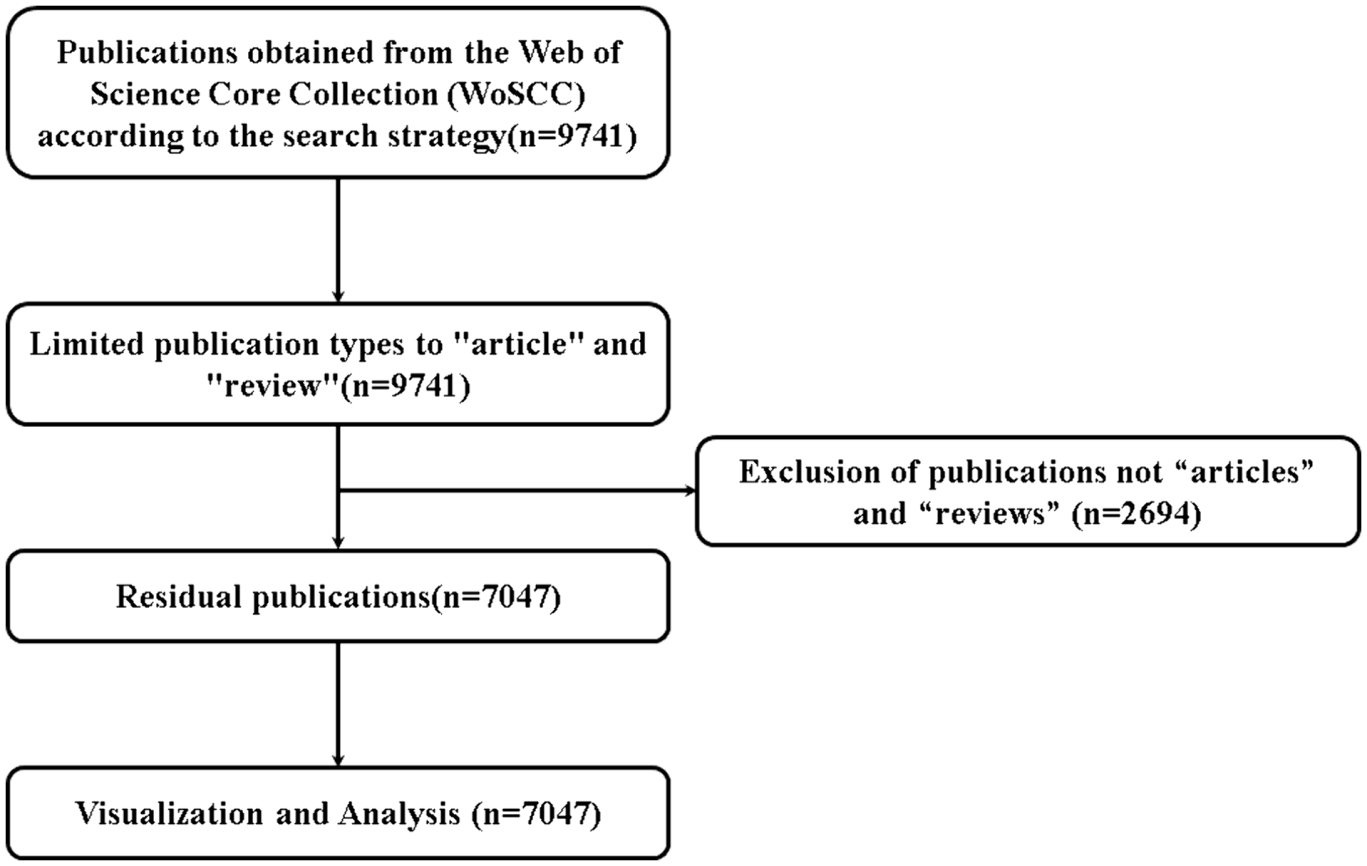
Literature screening flowchart for web of science core collection (WoSCC).

**Figure 2:**
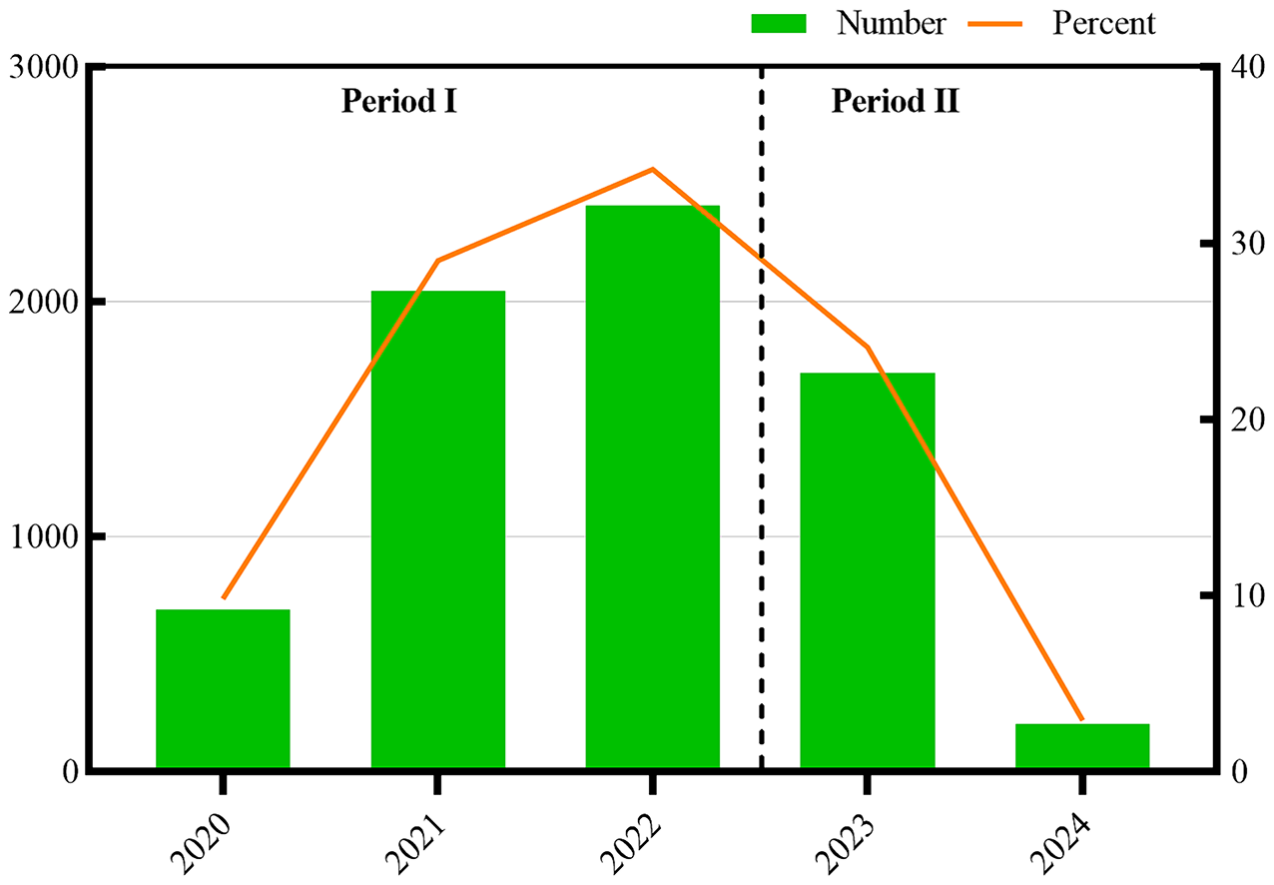
Annual research results on mental health during and post COVID-19 pandemic.

A total of 7,047 related publications were included including 6,548 ARTICLES and 499 REVIEWS (Figure 1). Based on the annual number of publications, we divided the entire period into two phases (Figure 2), the first phase (2020–2022), COVID-19 exploded since the end of 2019, the earliest related publications were seen in 2020, and then increased year by year, and 2022 possessed the highest amount of publications per year, with a total of 2,409 associated publications; In the second phase (2023–2024), the overall annual publications decreased year by year since 2023; due to the impact of normalized outbreak prevention and control, we predict that the number of publications in 2024 will continue to be lower than that in 2023.

### 3.2 Country and institutional analysis

**Figure 3:**
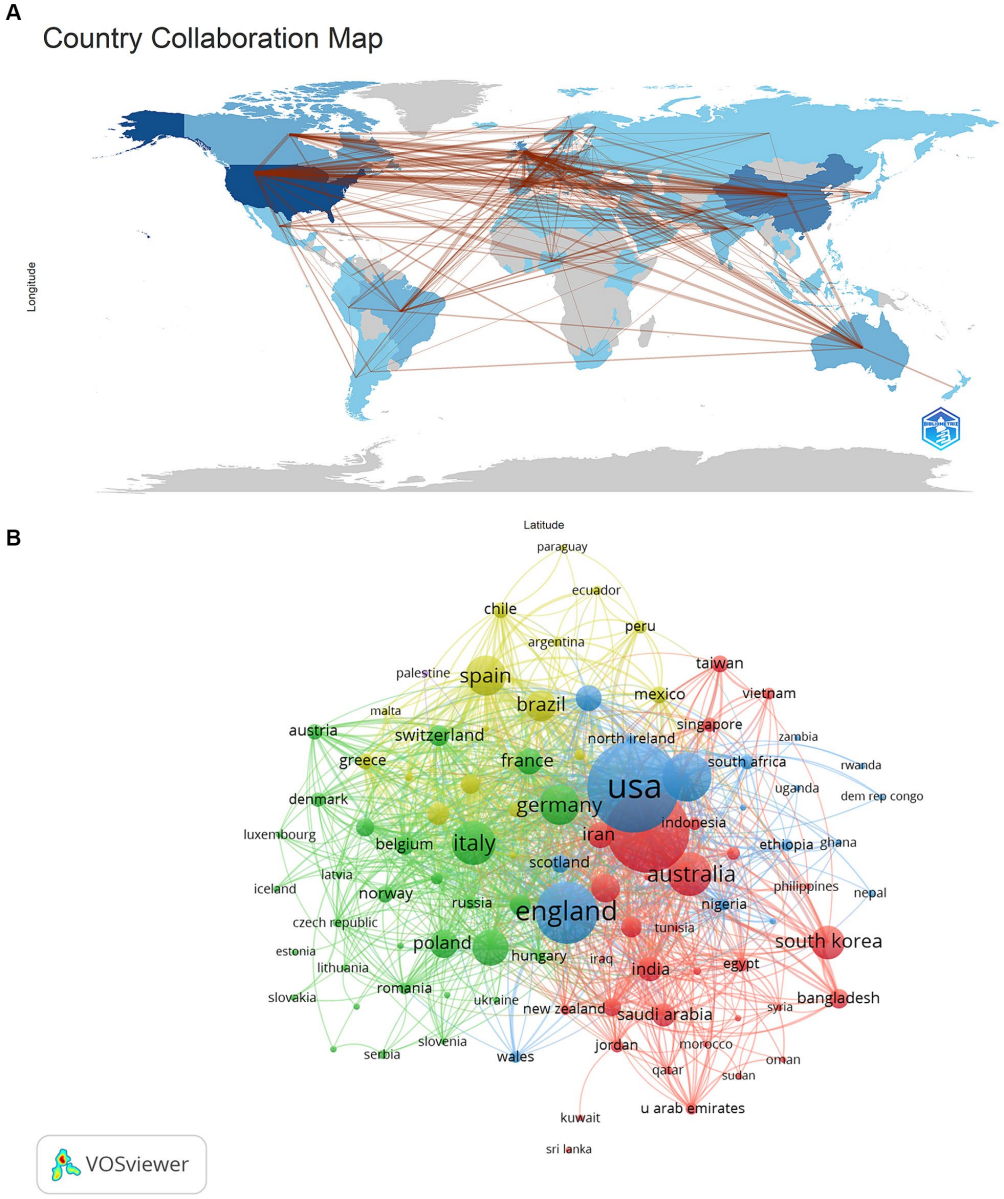
The country cooperation map **(A)** and visualization **(B)** on mental health during and post COVID-19 pandemic.

These publications came from 110 countries and 5,571 institutions; the top 10 countries were distributed among China, the United States, the United Kingdom, Canada, Italy, Turkey, Australia, Korea, Spain, and Germany, with a significant concentration in China and the United States (Table 1), and with China (1,199, 17.01%) and the United States (*n* = 1,003, 14.23%) accounted for almost 1/3 of the total number of publications; we filtered the countries with a several of publications greater than or equal to 15 and created a visualization network (Figure 3), and we found that there was more active cooperation between different countries, such as China, the United States, Canada and the United Kingdom, etc. (Figure 3).

**Table 1:** Top 10 countries and institutions on research of mental health during and post COVID-19 pandemic.

**Country**	**Articles**	**Affiliation**	**Articles**
China	1,199	University of Toronto	496
USA	1,003	University of London	462
United Kingdom	434	Harvard University	360
Canada	359	King’s College London	247
Italy	309	University College London	247
Turkey	266	University of California System	229
Australia	245	University of Melbourne	174
Korea	242	Institut National De La Sante Et De La Recherche Medicale (Inserm)	160
Spain	240	Mcgill University	156
Germany	238	University of Ottawa	155

**Figure 4:**
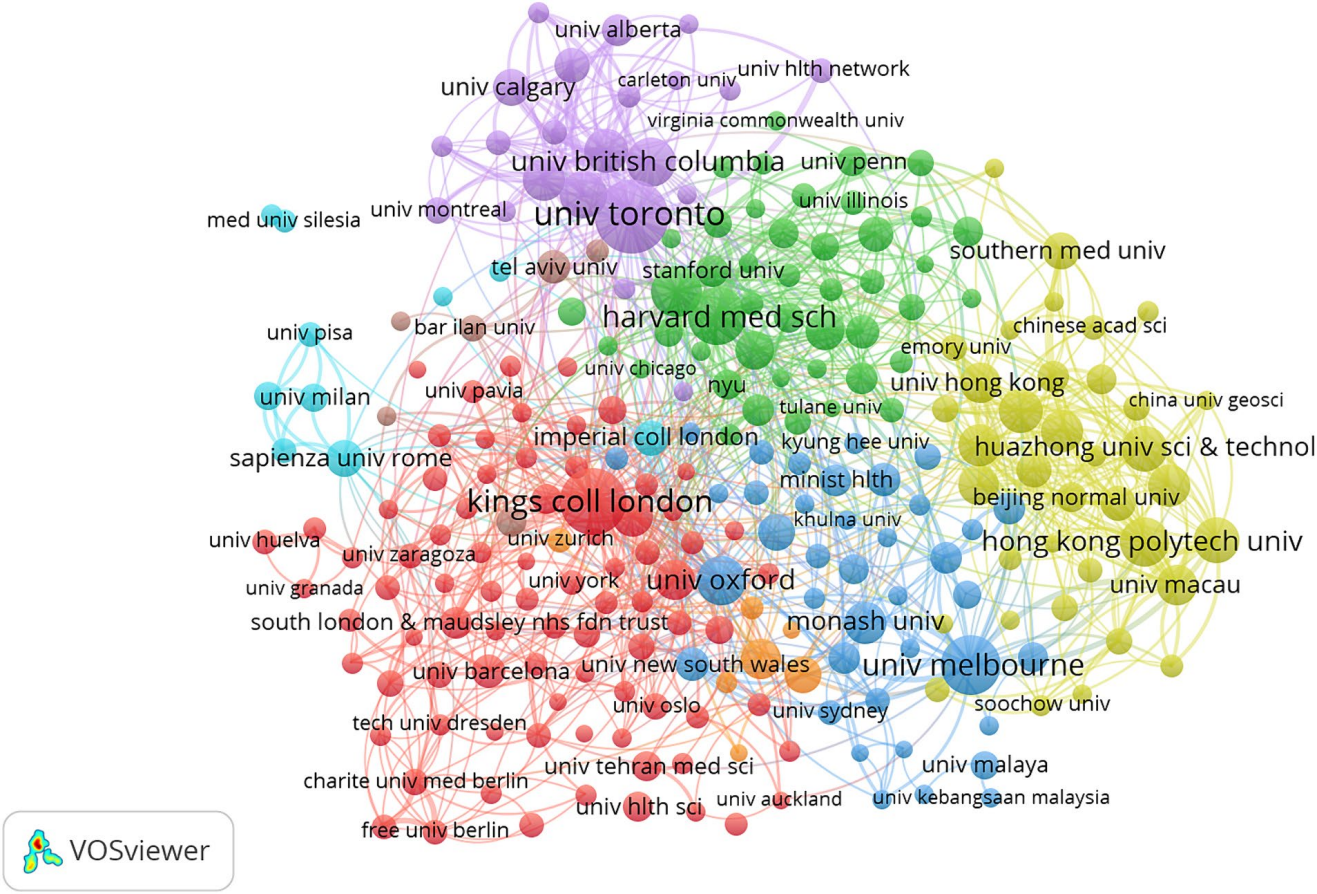
The visualization of institution on mental health during and post COVID-19 pandemic.

The top 10 institutions in terms of publications are located in 5 countries, with 3 from the United Kingdom and 3 from Canada, respectively University of Toronto, University of London, Harvard University, King’s College London, University College London, University of California System, University of Melbourne, Institut National De La Sante Et De La Recherche Medicale (Inserm), Mcgill University, University of Ottawa. Next, we visualized institutions with a minimum publication count of 15 (Figure 4), and studies were mainly focused on University of Toronto, King’s College London, University of Melbourne, Harvard University, Sichuan University, with more active collaboration within each center and a more even distribution overall.

### 3.3 Journals and co-cited journals

**Figure 5:**
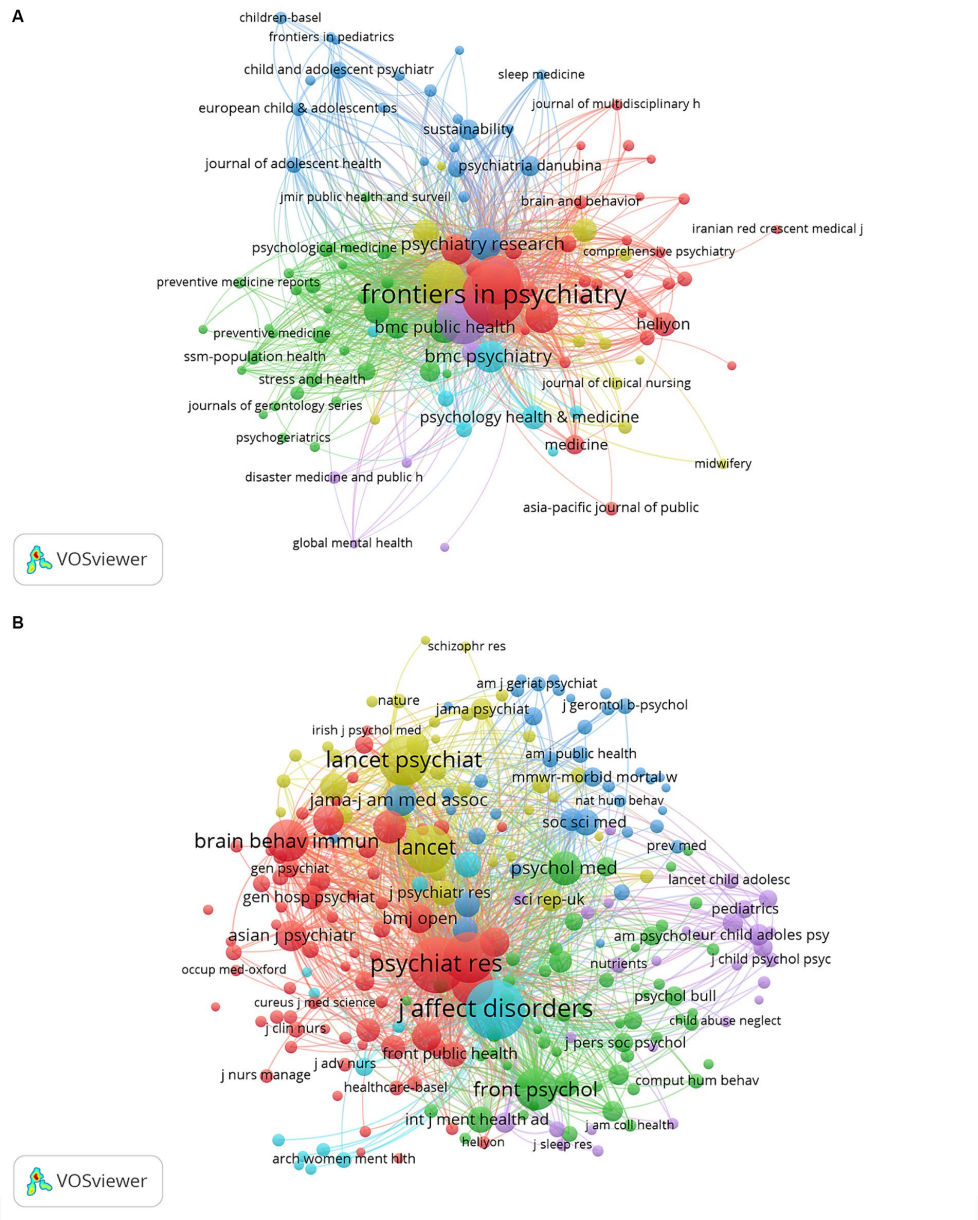
The visualization of journals **(A)** and co-cited journals **(B)** on mental health during and post COVID-19 pandemic.

These studies were published in 992 journals, with Frontiers in Psychiatry publishing the most significant number of publications (*n* = 481, 6.83%), followed by Frontiers in Public Health (*n* = 325, 4.61%), PLoS One (*n* = 285, 4.04%), Journal of Affective Disorders (*n* = 252, 3.58%), and Psychiatry Research (*n* = 135, 1.92%). Among the top 15 journals with the most engaged publications, the journals with the highest impact factor referring to the impact factor published by WOS 2022 were Psychiatry Research (IF = 11.3), Journal of Affective Disorders (IF = 6.6), and BJPsych Open (*n* = 5.4) (Table 2), Frontiers in Public Health (IF = 5.2) and Journal of Psychiatric Research (IF = 4.8). Subsequently, we visualized the 175 journals with a several of publications greater than or equal to 5 (Figure 5A). Figure 5A shows Frontiers in Psychiatry has active citation relationships with journals such as Psychiatry Research, BMC Psychiatry, and Heliyon.

**Table 2:** Top 15 journals and co-cited journals for research on mental health during and post COVID-19 pandemic.

Rank	Journal	Count	IF	*Q*	Co-cited journal	Co-citation	IF	*Q*
1	Frontiers in Psychiatry	481	4.7	2	Journal of Affective Disorders	7,576	6.6	1
2	Frontiers in Public Health	325	5.2	1	Psychiatry Research	6,790	11.3	1
3	PLoS One	285	3.7	2	PLoS One	5,568	3.7	2
4	Journal of Affective Disorders	252	6.6	1	Lancet Psychiatry	5,149	64.3	1
5	Psychiatry Research	135	11.3	1	Lancet	4,837	168.9	1
6	Healthcare	130	2.8	2	Frontiers in Psychiatry	3,828	4.7	2
7	BMC Psychiatry	120	4.4	2	Brain Behavior and Immunity	3,740	15.1	1
8	BMC Public Health	119	4.5	2	Frontiers in Psychology	3,676	3.8	1
9	BMJ Open	118	2.9	2	Psychological Medicine	2,503	6.9	1
10	Scientific Reports	87	4.6	2	JAMA Network Open	2,358	13.8	1
11	Journal of Psychiatric Research	78	4.8	2	JAMA-Journal of The American Medical Association	2,151	120.7	1
12	Heliyon	73	4	2	Asian Journal of Psychiatry	2052	9.5	1
13	Journal of Clinical Medicine	71	3.9	2	BMJ-British Medical Journal	2019	105.7	2
14	Psychology Health and Medicine	68	3.8	2	BMC Public Health	1894	4.5	2
15	BJPsych Open	58	5.4	1	BMC Psychiatry	9,132	4.4	2

**Figure 6:**
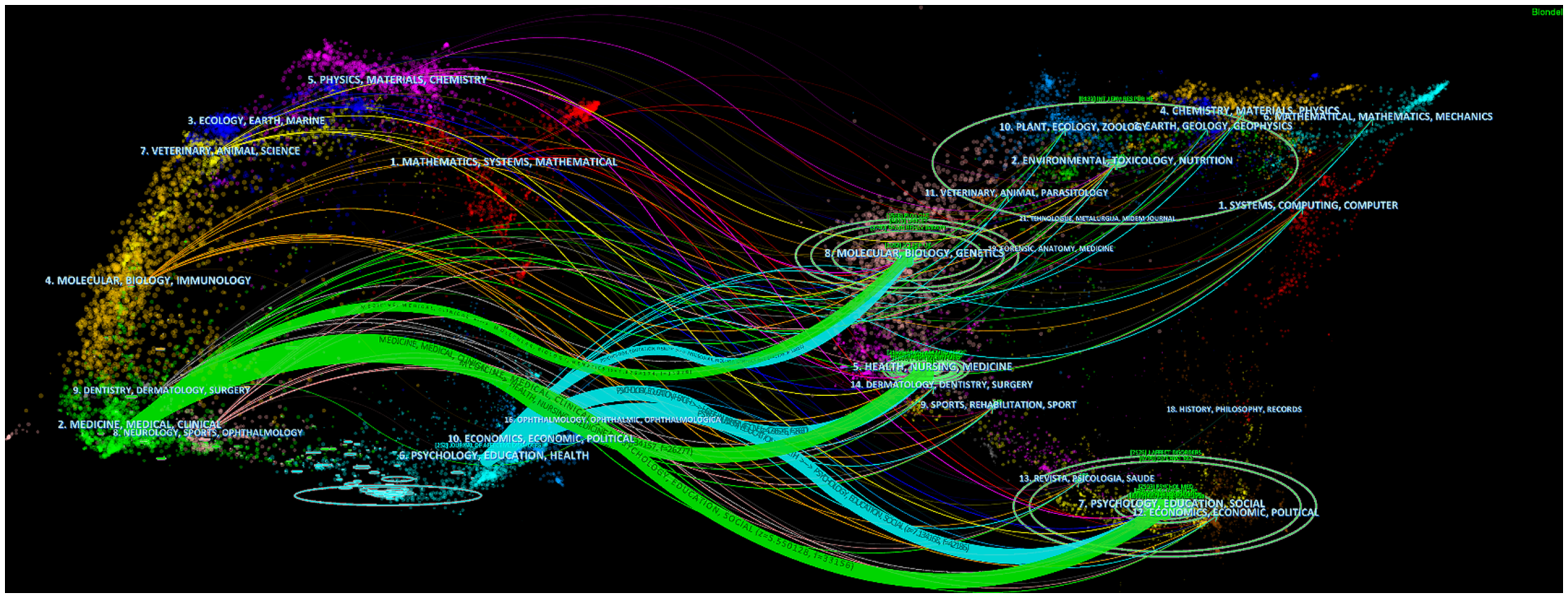
The dual-map overlay of journals on research mental health during and post COVID-19 pandemic.

Among the top 10 journals with the highest co-citation frequency had more than 2,000 co-citations (Table 2), and the most cited journal was Journal of Affective Disorders (co-citation = 7,576), followed by Psychiatry Research (co-citation = 6,790), PLoS One (total citations = 5,568), Lancet Psychiatry (total citations = 5,149), and Lancet (total citations = 4,837). In addition, the highest impact factor was found in Lancet (IF = 168.9), followed by JAMA-Journal of The American Medical Association (IF = 120.7), BMJ-British Medical Journal (IF = 105.7), Lancet Psychiatry (IF = 64.3) and Brain Behavior and Immunity (IF = 15.1). The 244 journals with a minimum number of co-citations more than 500 were screened to map the co-citation network (Figure 5B), Journal of Affective Disorders with Psychiatry Research, Frontiers in Psychiatry, Brain Behavior and Immunity, Frontiers in Psychology, etc. had a positive co-citation relationship. The dual-map overlay is showing the citation relationship between journals and co-cited journals. As shown in Figure 6, the green route is the primary citation route, which represents that the research results published in “2. MEDICINE/MEDICAL/CLINICAL” journals are mainly cited by “7. PSYCHOLOGY/EDUCATION/SOCIAL,” “5. HEALTH/NURSING/MEDICINE” and “8. MOLECULAR/BIOLOGY/GENETIC.”

### 3.4 Authors and co-cited authors

**Figure 7:**
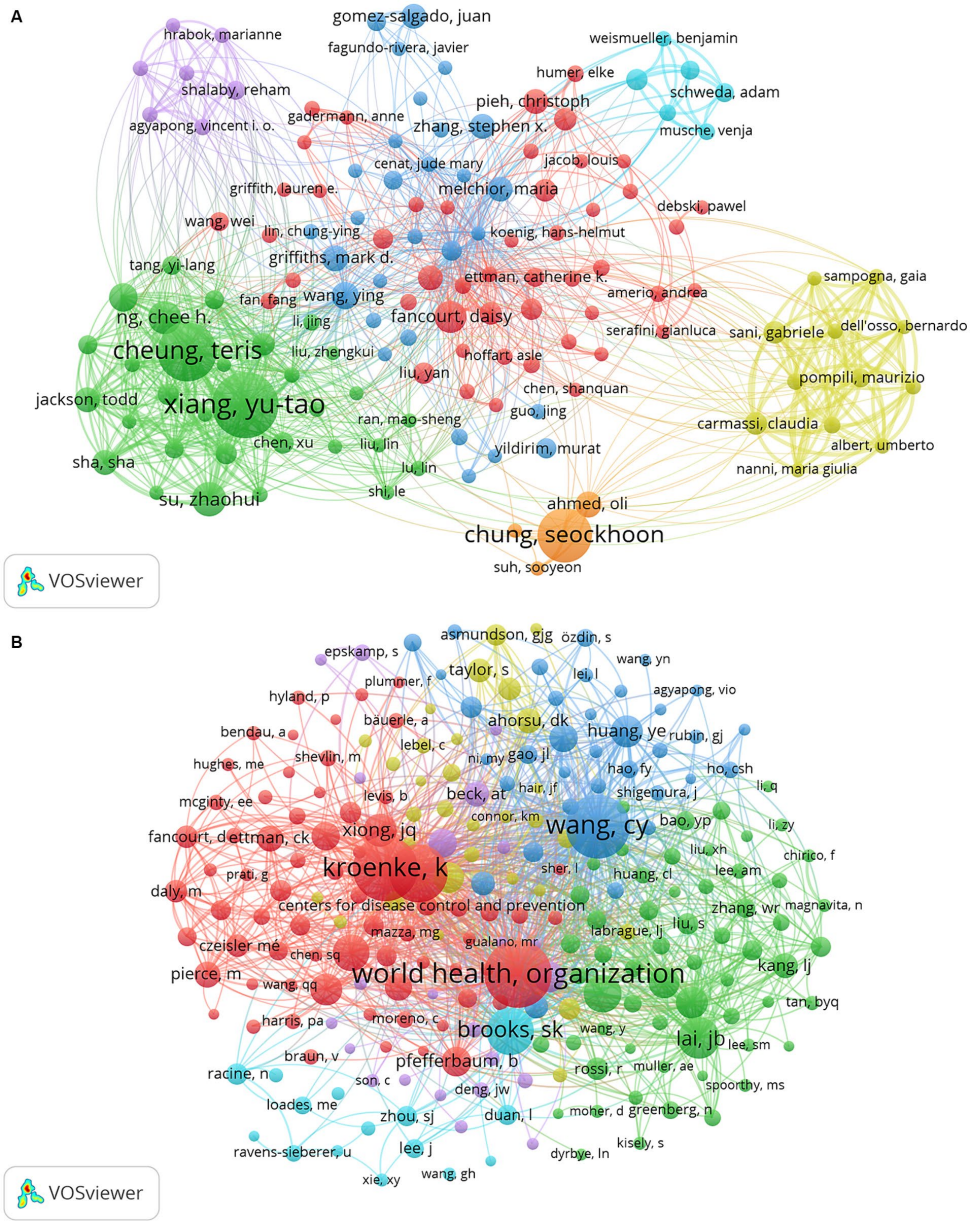
The visualization of authors **(A)** and co-cited authors **(B)** on research of mental health during and post COVID-19 pandemic.

A total of 36,486 authors participated in the study on mental health during and after the COVID-19 pandemic. When the authors were sorted by number of publications, the lowest number of publications among the top 10 authors was 16. We constructed a collaborative network map for the 137 authors with more than 8 publications (Figure 7A), and We found that Xiang Yu-Tao has the most significant node because he has the most publications, followed by Cheung Teris, Chung Seockhoon, Su Zhaohui, and NG Chee H. In addition, we have observed collaboration between multiple authors nearby. For example, Xiang Yu-Tao worked closely with Cheung Teris, Li Wen, and Su Zhaohui; Chung Seockhoon worked closely with Wang Ying, Ho Roger, and others, and Fancourt Daisy worked closely with Shi Li, Steptoe Andrew, and others. Seventy-six authors had more than 200 co-citations (Table 3), with the highest number being World Health Organization (*n* = 2,436), followed by Kroenke K (*n* = 1,678) and Wang CY (*n* = 1,618). We plotted the co-citation network map for the 234 authors with co-citations of more than 100 (Figure 7B), and we found that co-citations were frequent among different authors, such as World Health Organization with Wang CY, Brooks SK, etc.

**Table 3:** Top 10 authors and co-cited authors on research of mental health during and post COVID-19 pandemic.

Rank	Authors	Count	Co-cited authors	Citations
1	Xiang, Yu-Tao	47	World Health, Organization	2,436
2	Cheung, Teris	41	Kroenke, K	1,678
3	Chung, Seockhoon	37	Wang, CY	1,618
4	Su, Zhaohui	22	Spitzer, RL	1,360
5	NG, Chee H.	21	Brooks, SK	985
6	Fancourt, Daisy	20	Lai, JB	823
7	Zhao, Yan-Jie	18	Holmes, EA	665
8	Wang, Ying	17	Xiong, JQ	563
9	Ahmed, Oli	16	Huang, YE	549
10	Griffiths, Mark D.	16	Pappa, S	542

### 3.5 Co-cited references

**Figure 8:**
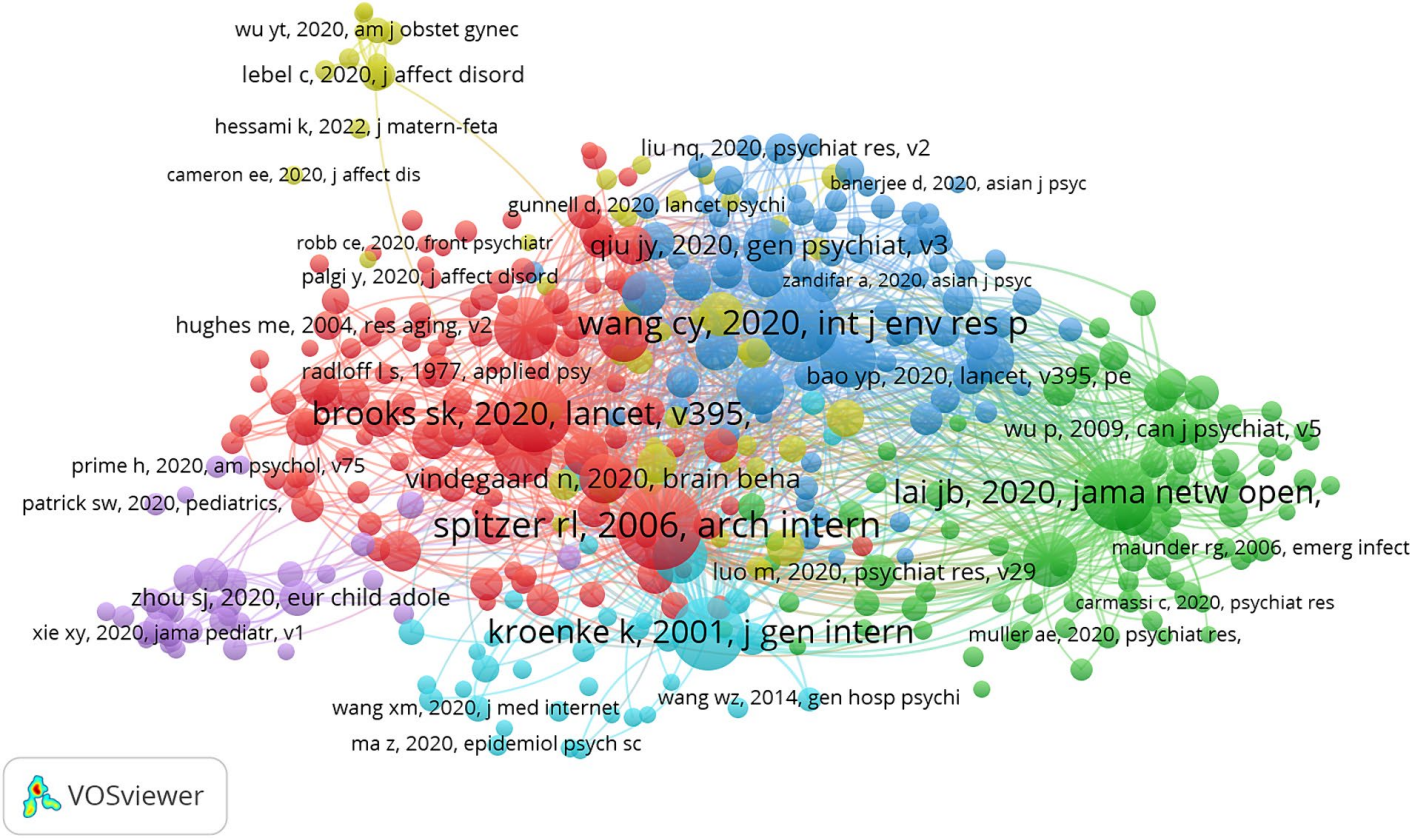
The visualization of co-cited references on research of mental health during and post COVID-19 pandemic.

Four hundred and six references have a co-citation frequency more significant than 50, and the minor co-citation frequency among the top 10 references is 49 (Table 4). We visualized the 406 references with a co-citation frequency more significant than 50 (Figure 8). We found that “Spitzer RL, 2006, Arch intern med” and “Wang CY, 2020, Int J Env Res Pub He” and “Brooks SK, 2020, Lancet” are more likely to be cited simultaneously.

**Table 4:** Top 10 co-cited references on research of mental health during and post COVID-19 pandemic.

Rank	Cited references	Citations
1	Spitzer RL, 2006, Arch Intern Med, V166, P1092, Doi 10.1001/Archinte.166.10.1092	1,166
2	Brooks Sk, 2020, Lancet, V395, P912, Doi 10.1016/S0140-6736(20)30460-8	865
3	Kroenke K, 2001, J Gen Intern Med, V16, P606, Doi 10.1046/J.1525-1497.2001.016009606.X	861
4	Lai Jb, 2020, Jama Netw Open, V3, Doi 10.1001/Jamanetworkopen.2020.3976	823
5	Holmes Ea, 2020, Lancet Psychiat, V7, P547, Doi 10.1016/S2215-0366(20)30168-1	658
6	Xiong Jq, 2020, J Affect Disorders, V277, P55, Doi 10.1016/J.Jad.2020.08.001	563
7	Huang Ye, 2020, Psychiat Res, V288, Doi 10.1016/J.Psychres.2020.112954	507
8	Pappa S, 2020, Brain Behav Immun, V88, P901, Doi [10.1016/J.Bbi.2020.05.026 10.1016/J.Bbi.2020.11.023]	500
9	Cao Wj, 2020, Psychiat Res, V287, Doi 10.1016/J.Psychres.2020.112934	493
10	Qiu Jy, 2020, Gen Psychiat, V33, Doi 10.1136/Gpsych-2020-100213	451

### 3.6 Reference with citation bursts

**Figure 9:**
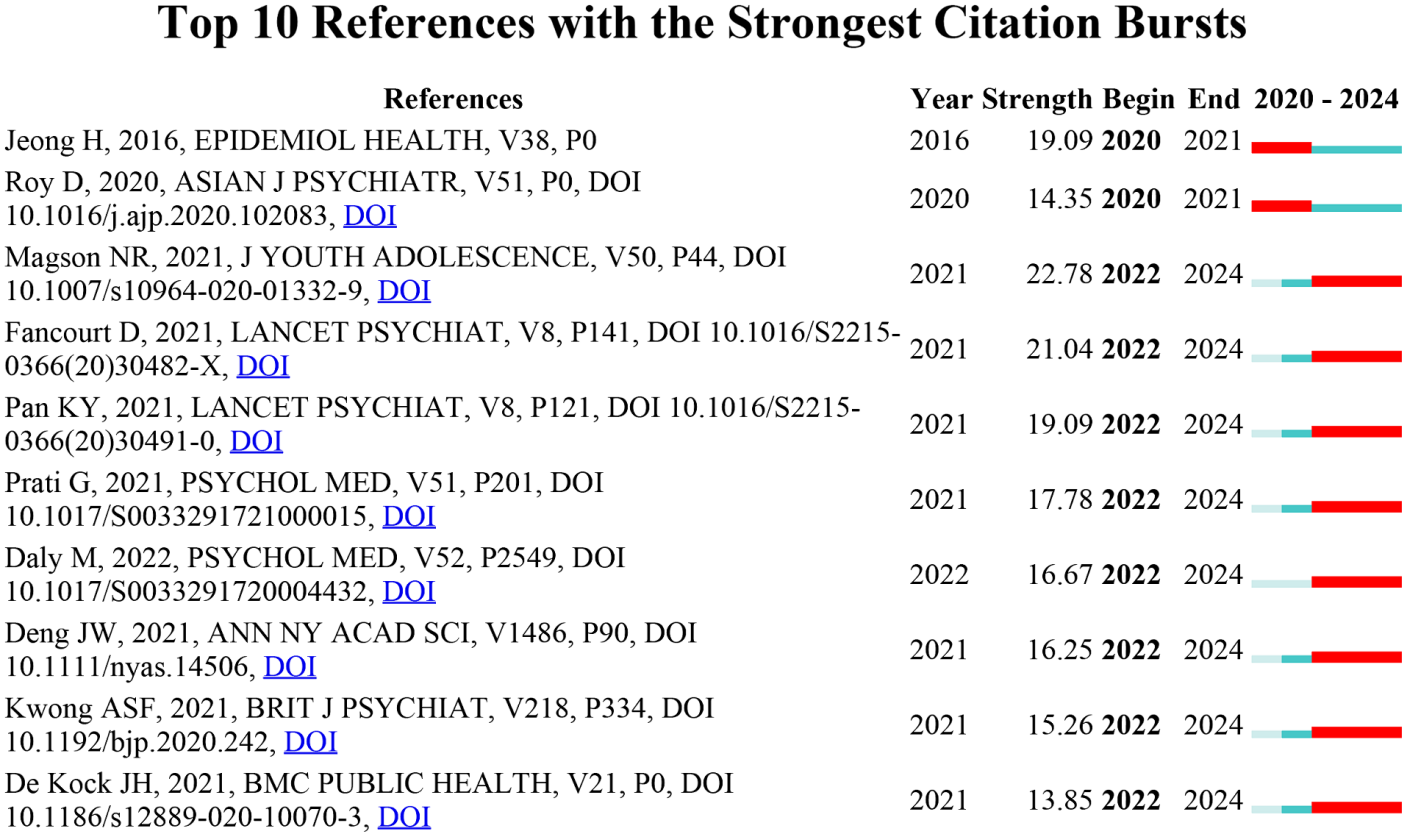
Top 10 references with strong citation bursts.

CiteSpace was used to identify 10 intense citation bursts (Figure 9), references that suddenly gained many number of citations in a given period, creating a significant peak in citation growth, with the years of substantial citations shown in red. The earliest reference with a considerable peak in citation growth was in 2021, and these bursts lasted about 2 years. The reference burst with the highest strength was “Magson NR, 2021, J YOUTH ADOLESCENCE, V50, P44, DOI 10.1007/s10964-020-01332-9” (Strength = 22.78), published by Magson NR et al. in J YOUTH ADOLESCENCE. The second was “Fancourt D, 2021, LANCET PSYCHIAT, V8, P141, DOI 10.1016/S2215-0366(20)30482-X, DOI” (Strength = 21.04), published by Fancourt D et al. In LANCET PSYCHIAT. The third was “Jeong H, 2016, EPIDEMIOL HEALTH, V38, P0” (Strength = 19.09), published by Jeong H et al. in EPIDEMIOL HEALTH. These 10 references have a burst strength range of 13.85–22.78 and a persistent strength of 1–2 years.

### 3.7 Hotspots and Frontiers

**Figure 10:**
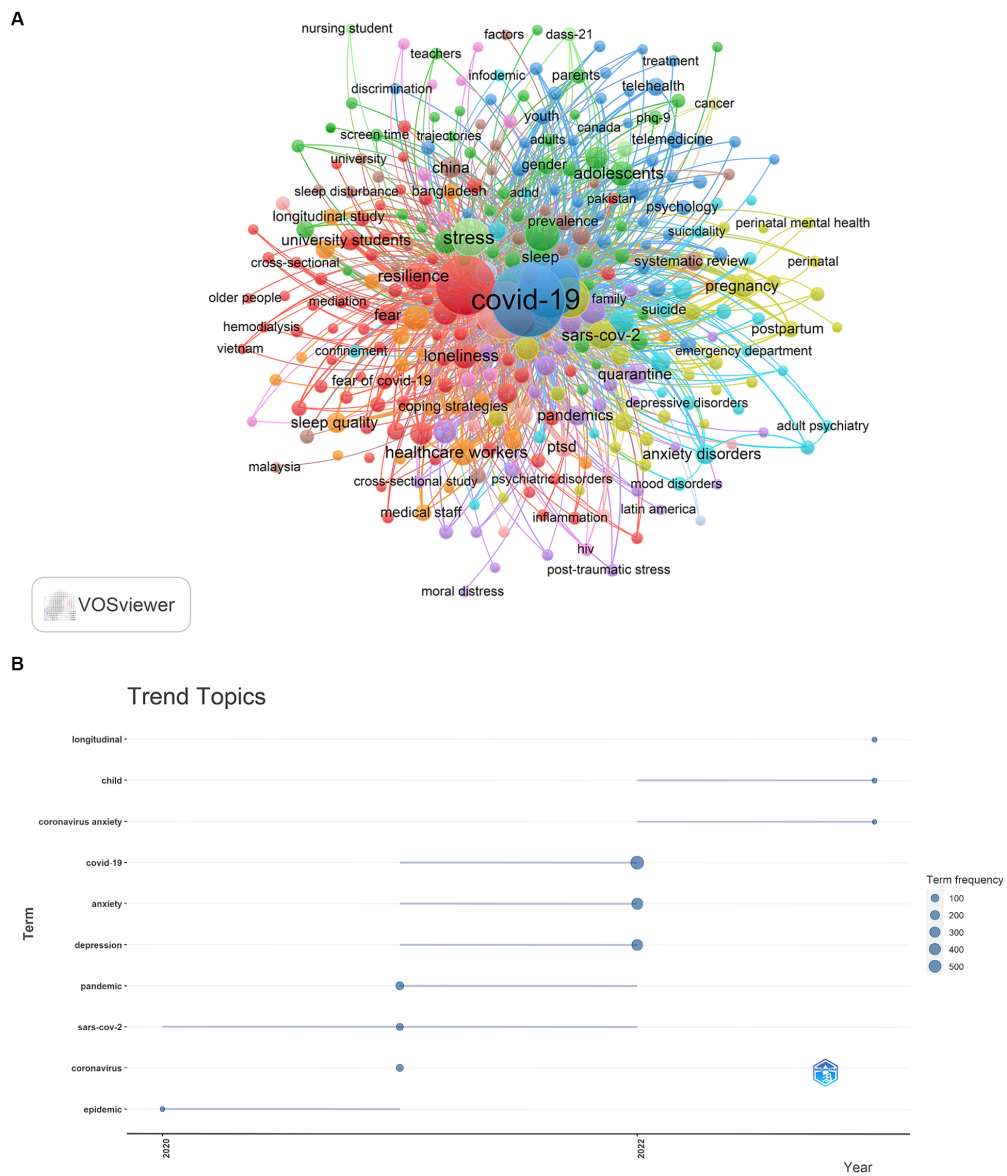
Keyword cluster analysis **(A)** and trend topic analysis **(B)** on research of mental health during and post COVID-19 pandemic.

The co-occurrence relationship between keywords can show research hotspots, development trends, and the degree of correlation between different topics. The top 20 keywords with the highest frequency all appeared more than 130 times (Table 5), and we visualized the keywords with more than 10 times (Figure 10). Based on the different colors of the nodes, we can classify them into three clusters, representing three research directions, which are clusters of epidemiology-related keywords such as COVID-19, pandemic, and covid-19 pandemic; mental health-related keywords such as mental health, anxiety, depression; and keyword clusters related to subjects such as workers, young adults, and adolescents.

**Table 5:** Top 20 keywords on research of mental health during and post COVID-19 pandemic.

Rank	Keywords	Counts	Rank	Keywords	Counts
1	COVID-19	4,317	11	Lockdown	181
2	Mental health	2,371	12	Depressive symptoms	170
3	Anxiety	2,170	13	Healthcare workers	169
4	Depression	2093	14	Adolescents	149
5	Pandemic	644	15	Public health	149
6	Stress	545	16	Loneliness	146
7	COVID-19 pandemic	411	17	Quality of life	144
8	Coronavirus	310	18	Insomnia	136
9	SARS-CoV-2	233	19	Pregnancy	131
10	Resilience	200	20	Social support	129

**Figure 11:**
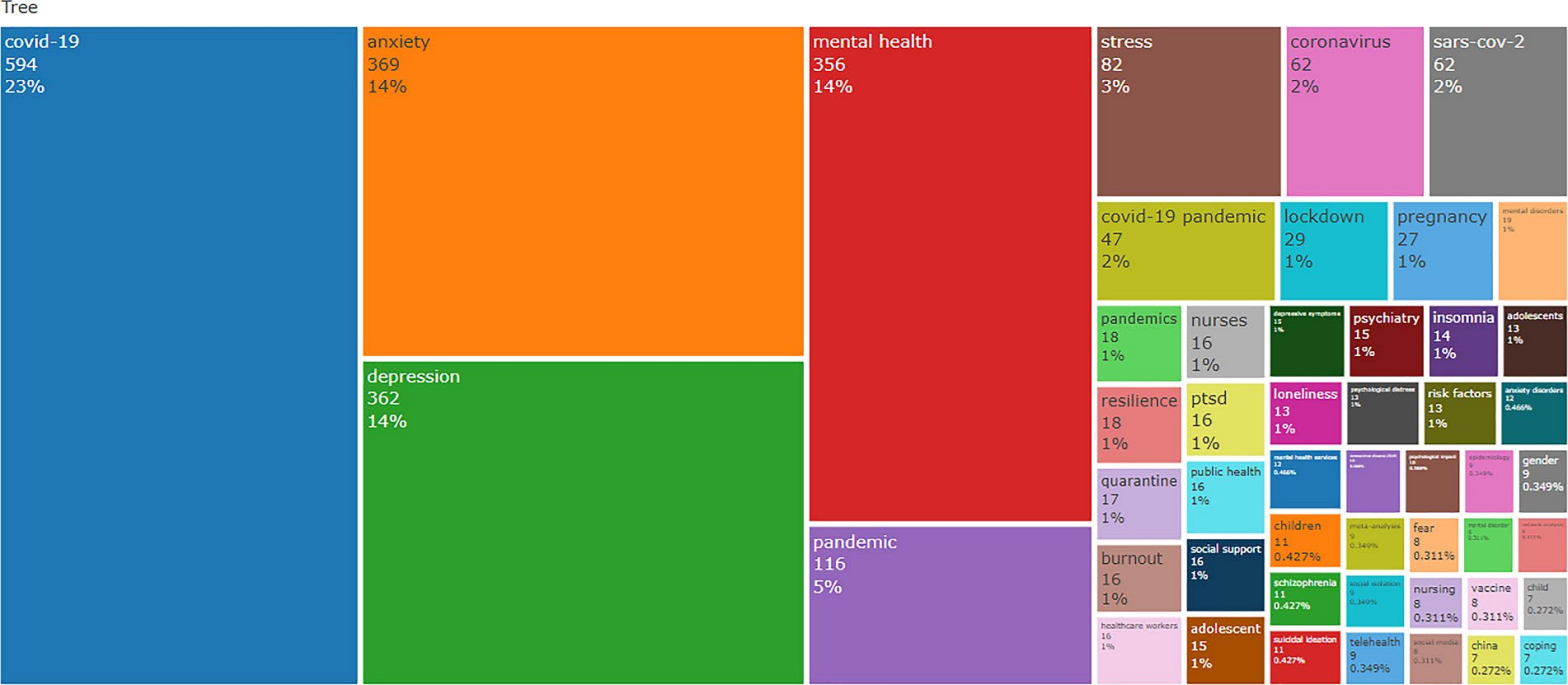
Keywords tree map of mental health during and post COVID-19 pandemic.

The R software (bibliometrix package, 3.2.1) analyzed the keywords and Treemap to show the trend themes (Figure 11), the relevant research started in 2020, and this period was mainly dominated by the keywords of pandemic, sars-cov-2, coronavirus, epidemiology, indicating that the relevant research was primarily focused on epidemiology-related research. After that, the keywords gradually focused on depression, coronavirus anxiety, anxiety, longitudinal and so on, indicating that the research direction gradually opened to focus on the mental state of COVID-19 patients, which includes the importance of mental health in the longitudinal period. In addition, the keywords longitudinal, child, and coronavirus anxiety appeared frequently in the past 2 years, so they probably represent the current hotspots of Mental Health During and Post COVID-19 Pandemic research.

## 4 Discussion

Mental health during and post COVID-19 pandemic has been an interesting topic over the past 5 years. Our study shows a growing body of literature on mental health during and post COVID-19 Pandemic, indicating this research’s growing popularity and importance. 7,047 publications related to Mental Health During and Post COVID-19 Pandemic were retrieved from the Web of Science database. The relevant publications were first seen in 2020 and finally increased year by year, with 2022 having the highest annual publications with 2,409 relevant publications. The overall yearly publications declined each year from 2023 onwards. The neocoronavirus pandemic sparked worldwide attention after the outbreak in early 2020, prompting many scientists and researchers to devote themselves to neocoronavirus-related research. At the pandemic’s beginning, the scientific community was highly active in research on neo-coronaviruses. As a result, a large number of research results may have been accumulated in 2020 and 2021, which may be published one after another in 2022, leading to a peak in the number of annual publications in addition to rapidly disseminating the results of the research on neo-coronaviruses, some academic journals may have taken measures to accelerate the review of manuscripts and publications, which also contributed to an increase in the number of annual publication volume in 2022. As time passes, the number of publications on coronaviruses decreases in 2023 and beyond; as the outbreak is controlled, knowledge of new coronaviruses increases, and concern about the outbreak fades.

China and the United States are the leading countries researching Mental Health During and Post COVID-19 Pandemic, with China ranking first. Among the top 10 research institutions, 30% were from the United Kingdom and 30% were from Canada, which tied for first place, and we noticed that there were more active collaborations among China, the USA, and the United Kingdom; regarding research institutions the University of Toronto, King’s College London, University of Melbourne, Harvard University and Sichuan University. Some countries have high rankings regarding the number of publications but have less cooperation. Therefore, we strongly recommend that there should be more in-depth cooperation between different institutions to promote the progress of Mental Health During jointly and Post COVID-19 Pandemic research.

Most of the findings related to Mental Health During and Post COVID-19 Pandemic have been published in journals under the broad category of PSYCHIATRY, indicating that this type of journals are the hottest journals. The top 15 journals are all in the Q1 and Q2 categories. The results for the co-cited journals were similar, and all of these journals also belonged to the broad categories of Q1 and Q2, these journals provide reliable evidence to support the Mental Health During and Post COVID-19 Pandemic study. Authors Xiang Yu-Tao, Cheung Teris, and Chung Seockhoon published most significant number of Mental Health During and Post COVID-19 Pandemic studies. The 10 most co-cited frequency references were selected, and these studies were focused on the year 2020, which provides the basis for subsequent analyses. References with citation bursts that suddenly gained a large number of citations in a given period, creating a significant peak in citation growth, focused on the aggregation of clinical evidence on Mental Health conditions across countries and subject types, as well as literature reviews. The results of co-occurrence relationship between keywords show that the Mental Health During and Post COVID-19 Pandemic research hotspots are mainly focused on Mental Health-related epidemiological studies and cohort studies, given that the persistence of the long COVID-19 will still need to be thoroughly explored. Keyword trends and topic results for the last 2 years showed that the frequency of longitudinal, child, and coronavirus anxiety was high, suggesting that child-related Mental Health issues need to be emphasized.

For the impact of the COVID-19 pandemic on mental health, national and international longitudinal studies and meta-analyses have shown a significant increase in psychological symptoms such as depression and anxiety (Prati and Mancini, 2021; Zhao et al., 2022). Despite differences in the prevalence and response to COVID-19 across countries, the early period of the 2020 pandemic in general showed a deterioration in overall mental health, a trend validated in international studies such as those by Findlay et al. (2020) in Canada, Fitzpatrick et al. (2020) in the United States, and Pierce et al. (2020) in the UK as shown in their studies. However, particular attention needs to be paid to the differences in the impact on countries at different income levels during a pandemic, and the mental health impact of COVID-19 might be more severe in low-and middle-income countries (Castro-de-Araujo and Machado, 2020), which may be related to inadequate health systems, poor preparedness, and economic instability in these countries. Demographic and socioeconomic characteristics have also been identified as risk factors for poor mental health outcomes. Although younger age groups face greater mental health risks at all times (i.e., outside of pandemic conditions), their mental health deterioration during COVID-19 appears to be more severe than that of older age groups (Biddle et al., 2024). This may be because age is associated with mental health risk factors and these may be exacerbated during a pandemic, such as instability in employment and financial status (Verick, 2009; Statistics ABo, 2021). Thus, although young people are relatively physically healthy, their mental health appears to be disproportionately affected (World Health Organization, 2020a). Another important risk factor identified is pre-existing mental health problems. For example, Galletly (2020) noted that a pandemic would be a difficult time for patients with chronic mental illness. Studies have shown that individuals with prior mental health diagnoses have poorer mental health during a pandemic.

Different trends have been shown for the impact of the COVID-19 pandemic on the psychological status and mental health of various populations (Zeverdegani, 2022). First, healthcare workers usually contract the disease through exposure to COVID-19-infected individuals, and a large number of studies have shown that the COVID-19 pandemic has increased mental health problems among healthcare workers (Rana et al., 2020). Studies of physicians have also demonstrated higher levels of depression, anxiety, and stress during the COVID-19 pandemic (Elbay et al., 2020); through surveys of nurses, depression, anxiety, and stress among nurses were at moderate levels during the COVID-19 pandemic (Abadi et al., 2020); and evaluations of medical students have shown that during the COVID-19 pandemic, the mild to severe anxiety and depression prevalence was high (Nakhostin-Ansari et al., 2020); and medical staff may face problems such as decreased sleep quality, which may exacerbate their psychological stress (Xiao et al., 2020). In similar pandemic scenarios, patients may have significant concerns, and studies on COVID-19 patients have shown some related psychological problems, with an increased prevalence of depression among COVID-19 patients (Zandifar and Badrfam, 2020; Zhang et al., 2020), and some emotional states, such as fear and anxiety, becoming the main effects faced by COVID-19 patients [6]. The results of epidemiologic studies in India have shown that a significant proportion of the general public suffered from moderate to very severe depression, anxiety and stress during the COVID-19 pandemic (Verma and Mishra, 2020; Rehman et al., 2021). During the COVID-19 pandemic, people had an increased prevalence of anxiety disorders (Huang and Zhao, 2020), and anxious people often have difficulty falling asleep. They may wake up frequently during the night (Xiao et al., 2020), which may affect the quality of sleep during the pandemic and proximally further exacerbate mental health conditions (Mo et al., 2020).

The COVID-19-associated increased risk of neuropsychiatric disorders was most pronounced during the first peak of the pandemic and decreased over the following 2 years (Taquet et al., 2021; Magnúsdóttir et al., 2022). As mentioned earlier, there are many reasons that can lead to COVID-19 mental health disorders, such as fear of death, stress of contracting a new pandemic disease, isolation and lack of social support, fear/guilt of transmitting COVID-19 to the family or community, and socio-economic hardship due to loss of wages (Zhang et al., 2022). COVID-19-associated neuropsychiatric disorders, such as fatigue, sleep disorders, and cognitive deficits commonly referred to by patients and clinicians as “brain fog,” manifest themselves differently in populations with varying degrees of infection severity, and also increase the risk of anxiety and depression to varying degrees (Dantzer et al., 2008). In reviewing the current literature, studies of autopsy specimens from patients who died of acute COVID-19 have shown severe hypoxic injury and neuroinflammatory neuropathologic manifestations in the brain, such as blood–brain barrier permeability accompanied by fibrinogen extravasation, microglia activation, and astrocyte hyperplasia (Cosentino et al., 2021; Thakur et al., 2021); And neuroimaging studies in acute COVID-19 patients have shown microstructural and functional changes in the hippocampus, a brain region critical to memory formation and the regulation of anxiety, emotion, and stress responses, which may explain a deeper level of mental health disorders in COVID-19 patients (Lu et al., 2020; Qin et al., 2021); Whereas, neuroimaging studies of the brains of patients after COVID-19 rehabilitation showed many changes in brain structure, such as a slight decrease in gray matter thickness in various regions of the cortex and the glia, diffuse edema, an increase in regional tissue damage markers related to olfactory cortex function, and a decrease in overall brain size (Tian et al., 2022), suggesting that the effects of COVID-19 on the patients’ long-term mental health effects after recovery cannot be ignored.

Overall, the impact of COVID is not limited to the duration of the epidemic, but may continue to affect people’s mental health for years or even longer. Mental health is equally as important as physical health, and we should pay particular attention and intervene early for those at the highest risk (Penninx et al., 2022). In future research, it is crucial to prioritize the following areas: (1) investigating the enduring effects of the pandemic on mental health over a longer period; (2) implementing early detection and prevention strategies for mental health problems in the aftermath of the pandemic, with a particular focus on children and adolescents. (3) facilitating the reintegration of individuals into society during the post-pandemic period. The specific aspects to be addressed are as follows: Most current research focuses primarily on the acute and short-term effects of pandemics on mental health, usually spanning a few months to a year of pandemic impact. However, longer follow-up of how pandemics affect population mental health and the impact on population mental health after a pandemic is also a focus for future research, with particular attention to the long-term effects on young people, as our study also confirms this. With mental health issues being particularly prominent, we need to take a series of measures to meet these challenges. First, providing mental health education is crucial. People need to understand the emotional reactions they may face and how to recognize and deal with them. Through education, people can better understand their mental health needs and know how to seek help when needed. Secondly, it is also crucial to build and strengthen social support systems. Establishing close ties with family, friends, and community can provide emotional support and comfort during tough times. Social support not only reduces an individual’s psychological stress, but also strengthens people’s resilience and helps them cope better with challenges. It is also essential to provide easily accessible mental health services, such as online mental health counseling services, mental health hotlines, and help from mental health professionals. In addition, advocating and supporting a healthy lifestyle is an essential strategy for dealing with mental health issues. Regular exercise, traditional Chinese Kung Fu (Baduanjin, Taiji, etc.), traditional Chinese medicine (acupuncture, moxibustion, tuina, etc.), yoga, music therapy, as well as a healthy diet and good sleeping habits can all help to alleviate anxiety and depression, and improve people’s mental health. Furthermore, the mental health needs of specific groups of people should be emphasized and attended to. For example, the medical and nursing staff, those who were unemployed or suffered pay cuts during the epidemic, the lonely older adults, and those with a history of mental illness may need exceptional support and attention.

Compared with similar previous studies (Chen et al., 2021), our study focused not only on mental health issues during the COVID-19 epidemic, but also on the long-term effects of the pandemic after its end, and this holistic perspective may have provided more profound insights into the evolution of mental health during and after the outbreak; in addition, we used a more comprehensive literature search strategy and analyzed more relevant publications, thereby minimizing publication selection bias, all of which may have increased the credibility and accuracy of the study. But there are still some limitations in this study, we only searched one database, Web of Science Core Collection (WoSCC), mainly because of the limitation of the bibliometric software Citespace and VOSviewer software, which can not merge multiple databases at the moment (especially in Chinese and English), and the quality of the publications in WoSCC is relatively high, and this database is a better representative choice. However, we have to recognize that ignoring other databases and non-English writing publications may result in some of the relevant publications being missed, there may be a potential bias.

## 5 Conclusion

We comprehensively analyzed 7,047 relevant publications, to identify the multidimensional aspects and scientific landscape related to mental health during and after the COVID-19 pandemic. This analysis contributes significantly to mental health research, particularly in guiding research directions and advancing knowledge post-pandemic. On the one hand, despite a reduction in the number of relevant studies in recent years, research on mental health during and after the COVID-19 pandemic remains of paramount importance, especially post-pandemic. On the other hand, given that mental health disorders may persist in the long term and the lingering effects of COVID-19 sequelae, it is crucial to pay special attention to specific populations, especially the mental health needs of children. Researchers in this field can use research findings to deepen understanding of post-COVID-19 mental health, identify appropriate research partners and sponsors, and gain insight into the latest relevant research findings that can guide future studies.

## Data availability statement

The original contributions presented in the study are included in the article/supplementary material, further inquiries can be directed to the corresponding author.

## Author contributions

LW: Conceptualization, Data curation, Funding acquisition, Writing – original draft, Writing – review & editing. LY: Software, Visualization, Writing – original draft. YJ: Software, Visualization, Writing – original draft. XP: Software, Visualization, Writing – original draft. XW: Conceptualization, Methodology, Software, Supervision, Validation, Writing – original draft, Writing – review & editing.

## Footnotes

^1^ https://www.bibliometrix.org
